# Altered Sox9 and FGF signaling gene expression in *Aga2* OI mice negatively affects linear growth

**DOI:** 10.1172/jci.insight.171984

**Published:** 2023-11-08

**Authors:** Jennifer Zieba, Lisette Nevarez, Davis Wachtell, Jorge H. Martin, Alexander Kot, Sereen Wong, Daniel H. Cohn, Deborah Krakow

**Affiliations:** 1 Department of Orthopaedic Surgery, David Geffen School of Medicine at University of California, Los Angeles, Los Angeles, California, USA.; 2 Department of Molecular Cell and Developmental Biology, University of California, Los Angeles, Los Angeles, California, USA.; 3 Department of Human Genetics, David Geffen School of Medicine at University of California, Los Angeles, Los Angeles, California, USA.; 4 Department of Psychology, University of California, Los Angeles, Los Angeles, California, USA.; 5 Department of Obstetrics and Gynecology and; 6 Department of Pediatrics, David Geffen School of Medicine at University of California, Los Angeles, Los Angeles, California, USA.

**Keywords:** Bone Biology, Development, Cartilage, Cell stress, Genetic diseases

## Abstract

Osteogenesis imperfecta (OI), or brittle bone disease, is a disorder characterized by bone fragility and increased fracture incidence. All forms of OI also feature short stature, implying an effect on endochondral ossification. Using the *Aga2^+/–^* mouse, which has a mutation in type I collagen, we show an affected growth plate primarily due to a shortened proliferative zone. We used single-cell RNA-Seq analysis of tibial and femoral growth plate tissues to understand transcriptional consequences on growth plate cell types. We show that perichondrial cells, which express abundant type I procollagen, and growth plate chondrocytes, which were found to express low amounts of type I procollagen, had ER stress and dysregulation of the same unfolded protein response pathway as previously demonstrated in osteoblasts. *Aga2^+/–^* proliferating chondrocytes showed increased FGF and MAPK signaling, findings consistent with accelerated differentiation. There was also increased *Sox9* expression throughout the growth plate, which is expected to accelerate early chondrocyte differentiation but reduce late hypertrophic differentiation. These data reveal that mutant type I collagen expression in OI has an impact on the cartilage growth plate. These effects on endochondral ossification indicate that OI is a biologically complex phenotype going beyond its known impacts on bone to negatively affect linear growth.

## Introduction

Osteogenesis imperfecta (OI), or brittle bone disease, is a genetically and phenotypically heterogeneous disorder resulting from mutations in at least 22 genes (1–3). More than 80% of OI cases result from dominantly inherited mutations in *COL1A1* and *COL1A2*, which encode the α1(I) and α2(I) chains of type I procollagen (3, 4). The products of the remaining OI-associated genes are involved in type I procollagen biosynthesis, posttranslational modifications, trafficking, cross-linking of telopeptide residues, osteoblast differentiation and function, and processes involved in bone mineralization. All forms of OI share the features of low bone mass, propensity to fracture, and short stature (4–11). While the severity of OI can be subdivided clinically into mild, moderate, severe, and perinatal lethal forms, all forms of OI are associated with growth deficiency, which is unrelated to the degree of fracture frequency. In the severe form of OI, extremely short stature is a consistent feature, with the average adult height of 92–108 cm (compared with 162 cm for female to 175 cm for male in average-stature individuals) (12). Multiple OI mouse models exhibit decreased body length, indicating that defective genes underlying OI have a negative effect on linear growth through the process of endochondral ossification (13–19). These observations raise the question of how cellular dysfunction resulting from the production of structurally abnormal type I collagen affects the cartilage growth plate (20–23).

Cartilage growth plate formation begins with early mesenchymal condensation, which differentiates into a chondrocytic lineage. Chondrocytes then proceed through the stages of proliferation, differentiation, hypertrophy, and then apoptosis or transdifferentiation into osteoblasts (24). Growth plate chondrocytes express high levels of type II collagen whereas the surrounding perichondrium, including the groove of Ranvier, express high levels of type I collagen. The periosteum and contiguous perichondrium surround the external surface of bone and cartilage, respectively, and contribute to skeletogenesis via signaling cascades and contribute to the reservoir of osteochondro-progenitor cells (25–29). These cells play specific roles in bone development and homeostasis, fracture healing, and growth plate dynamics (30). Molecular signaling communication between the perichondrium and growth plate cells is essential to maintaining growth plate dynamics and proper bone formation (28, 31). Hedgehog (IHH), TGF-β, WNT, and FGF are among the main communication pathways used by the perichondrium to direct endochondral growth (32). Parathyroid hormone–related protein (PTHrP) from the perichondrium acts on chondrocytes and maintains their proliferation, while induced hedgehog signaling promotes chondrocyte differentiation as well as increases PTHrP expression (33). FGF ligands expressed by the perichondrium are highly influential on growth plate development specifically by decreasing chondrocyte proliferation while delaying terminal chondrocyte hypertrophy (34). The interplay of the perichondrium and growth plate chondrocytes are key to coordinated endochondral ossification.

Collagen fibrils are the principal source of tensile strength in numerous tissues, and properly synthesized and cross-linked collagen is fundamental to skeletal integrity. Type I procollagen (product of *COL1A1* and *COL1A2*) is cotranslationally translocated into the lumen of the endoplasmic reticulum (ER), and numerous molecular chaperones and enzymes assist in its posttranslational modification, trimerization, maturation, and secretion (35). Genetic alterations have varying effects on osteoblast differentiation and function. With type I collagen mutations, osteoblast numbers are increased but exhibit delays in terminal differentiation and increased plasticity toward an adipocyte lineage (36). In addition, OI osteoblasts exhibit decreased synthesis and matrix incorporation of type I collagen (37–40). Mutant or overmodified type I collagen fibrils cause ER stress and the upregulation of the unfolded protein response (UPR) in fibroblasts and osteoblasts in both mouse models and patient-derived cells (18, 19, 41–45). Because of perichondrial type I collagen expression, synthesis of abnormal collagen could alter its cellular function, resulting in the disruption of signaling mechanisms that control skeletal progenitor differentiation, osteoblast numbers and function, and growth plate dynamics. We have previously shown that the inhibition of ER stress in an OI mouse model results in increased long bone growth, supporting the hypothesis that ER stress due to type I collagen mutations influences growth plate development (46).

In this study we used the *Aga2^+/–^* (*Col1a1^+/–^* c.-16 T>A, exon 50) mouse model to determine the molecular mechanisms underlying short stature in OI. This model recapitulates OI — the mice have low bone mass, spontaneous fractures, and decreased body length — and is an established OI model to study ER stress. We performed single-cell RNA-sequencing (scRNA-Seq) analysis of wild-type (WT) and *Aga2^+/–^* mouse cartilage tissues. Our analyses from WT cartilage showed extensive chondrocyte and perichondrial cell heterogeneity based on gene expression. This analysis expands our understanding of chondrocyte biology and provides a resource for future growth plate studies. We found, similar to osteoblasts derived from this model, ER stress via the protein kinase R-like ER kinase (PERK) arm of the UPR was upregulated in both perichondrial and growth plate cells, uncovering an unappreciated mechanism that mutant type I collagen induced ER stress and impacted progenitor cells. We observed growth plate dysregulation of signaling pathways associated with endochondral growth, with FGF and MAPK signaling being the most substantial; these alterations would lead to accelerated differentiation of proliferative growth plate chondrocytes and negatively affect growth. Furthermore, *Sox9* expression in chondrocyte tissues was increased, including inappropriate or ectopic *Sox9* expression in the hypertrophic zone, which would also contribute to accelerated proliferation and would negatively impact terminal differentiation. These data show that OI due to mutant type I collagen has an impact on the cartilage growth plate resulting from accelerated chondrocyte differentiation, alterations of progenitor cell development, increased FGF/MAPK signaling, and altered *Sox9* expression, all of which negatively affect the process of endochondral ossification and affect linear growth.

## Results

### Postnatal Aga2^+/–^ growth plates show statistically shorter proliferative zones.

**Figure 1:**
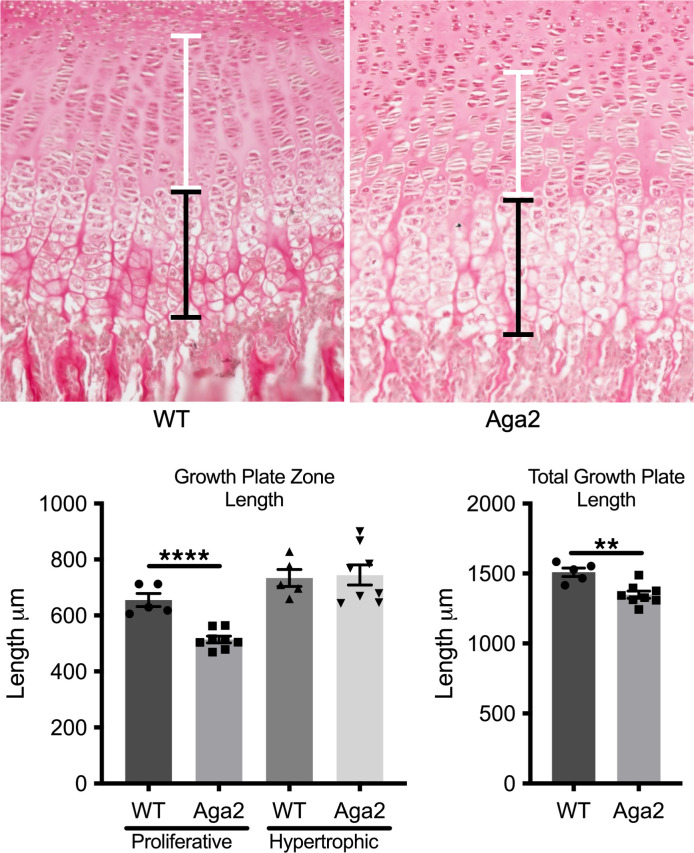
*Aga2^+/–^* growth plate abnormalities. WT and *Aga2^+/–^* P8 growth plate sections stained with Picrosirius red and imaged at 20× original magnification. Quantification of proliferative (white bar) and hypertrophic (black bar) zones indicates a significantly decreased proliferative zone length in *Aga2^+/–^* mice with no change hypertrophic zone length. WT *n* = 5, *Aga2^+/–^* *n* = 8. Data represent mean ± SEM. ** = *P* < 0.01, **** = *P* < 0.0001.

The *Aga2^+/–^* mouse contains a single base pair insertion that disrupts a splice site resulting in an extension of the COL1A1 protein (*Col1a1^+/–^* c.-16 T>A, exon 50) (18). This model is a validated OI mouse model and used to study ER stress as a mechanism of disease. Histologic evaluation of the *Aga2^+/–^* postnatal day 8 (P8) mouse growth plates stained with Picrosirius red showed statistically significant shortening of the proliferative zone with no change in the hypertrophic zone length (Figure 1). This suggests alterations in either chondrocyte proliferation (decreased) or differentiation (accelerated) possibly resultant from effects on progenitor cell recruitment and differentiation (47).

### ScRNA-Seq profiling of P5 mouse cartilage growth plate tissues.

To determine the molecular changes behind the *Aga2^+/–^* abnormal growth plate, we isolated mouse P5 growth plate tissues from both the distal femur and proximal tibia of WT and *Aga2^+/–^* mice after manually and enzymatically removing muscle and tendon tissue while keeping the perichondrium intact. This time point is prior to the development of the secondary ossification center. Cartilage tissues were dissociated using a collagenase and trypsin digestion before applying the Chromium single-cell 3′ library construction platform to capture approximately 10,000 cells per sample. Five mice were included in each group (WT and *Aga2^+/–^*), with 4 females and 1 male in the WT group, and 2 females and 3 males in the *Aga2^+/–^* group. We did not find sex-specific expression changes in our analyses. An average of 3.8 million reads were sequenced with an average of 41,000 reads per cell and over 20,000 genes mapped in an estimated total of 80,486 cells from all 10 samples. WT samples were collected and analyzed first, and *Aga2^+/–^* samples were collected and analyzed on a different day.

### Single-cell analysis shows high levels of cell heterogeneity in cartilage growth plate zones.

**Figure 2:**
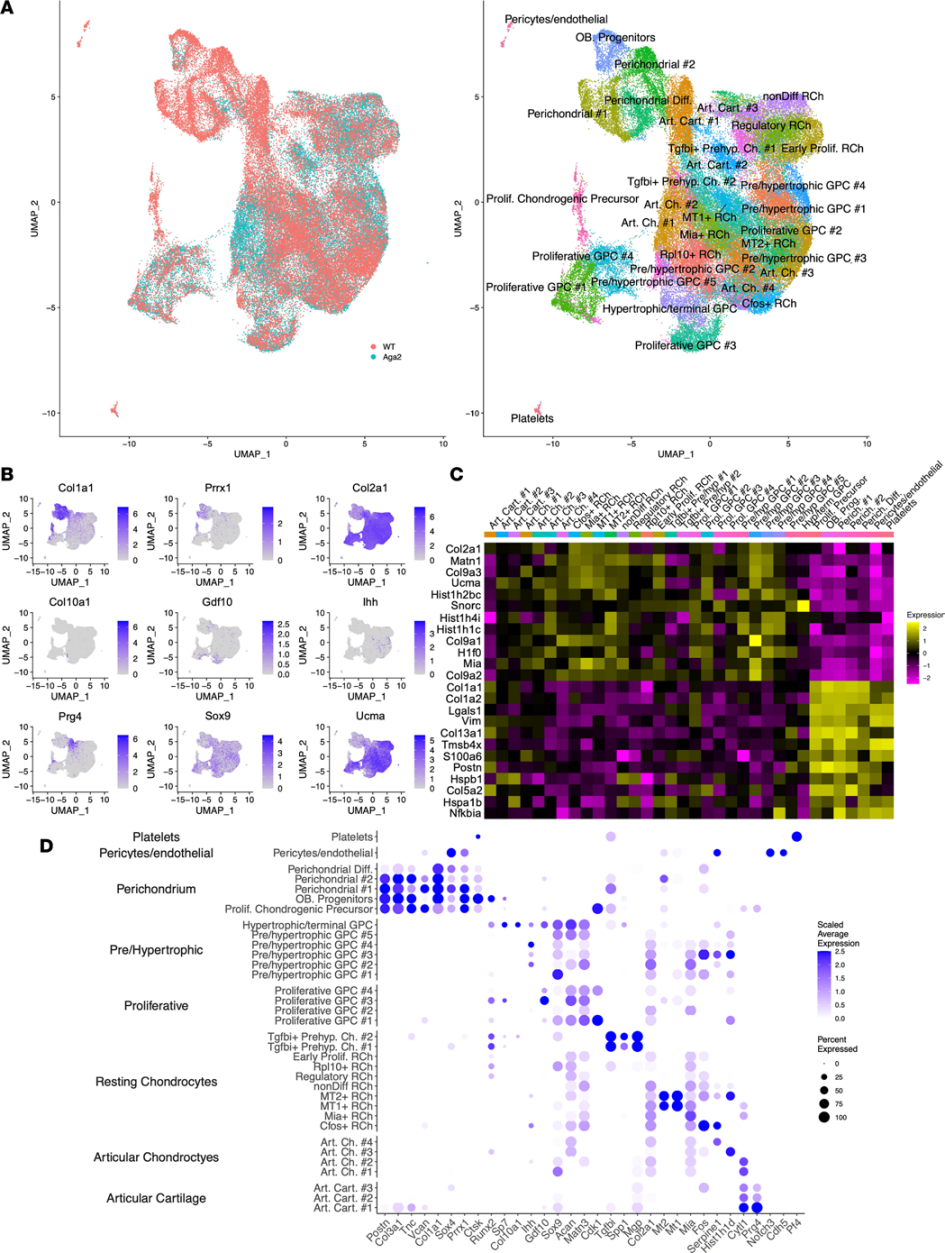
ScRNA-Seq analysis of P5 WT and *Aga2^+/–^* growth plates. (**A**) Cell clusters from scRNA-Seq analysis visualized by uniform manifold approximation and projection (UMAP). Left: Cells that correspond to WT (pink) and *Aga2^+/–^* (blue) growth plates. Right: Colors indicate clusters of various cell types. *N* = 5 for each genotype. (**B**) Feature plots showing the expression of multiple chondrocyte markers. (**C**) Heatmap showing top differentially expressed genes in each cluster. (**D**) Dot plot showing the expression of selected markers of various cell types. Dot size represents the percentage of cells expressing a specific marker, while the intensity of color indicates the average expression level for that gene, in that cluster.

Historically, growth plate chondrocytes were categorized into articular, resting, proliferative, prehypertrophic, and hypertrophic all surrounded by perichondrial cells. Recently, however, single-cell studies have begun to recognize higher levels of cellular heterogeneity than previously appreciated, but the full extent of cellular heterogeneity has not been fully described (48–52). Using the Seurat package, we performed principal component analysis and k-means clustering following quality control filtering, subsetting, and normalization. Cells were clustered based on their RNA expression profiles, and uniform manifold approximation and projection (UMAP) cluster analysis of the integrated single-cell data revealed 34 unique clusters (Figure 2A). Cell types were annotated based on extensive literature searches of published cartilage single-cell data and previously published RNA expression in situ studies (48–52). Clusters were initially categorized into 4 broad cell types: resting, articular, differentiating, and perichondrial. This was based on the top marker genes for each cluster when globally compared with all other clusters in the data set. For example, clusters in which proteoglycan 4 (*Prg4*) was the top differentially expressed gene when compared with all other clusters were categorized as articular (Figure 2, B–D). To further delineate between cell subtypes, the clusters were further subdivided from the broad categories and analyzed again to identify top marker genes when locally compared with other clusters within the same category. For example, when compared to all clusters, resting chondrocytes expressed matrillin 3 (*Matn3*) as a top differentially expressed gene, yet within resting chondrocyte clusters, the cluster designated as MT2+ RCh had metallothionein 2 (*Mt2*) as the top differentially expressed gene in comparison with the other resting chondrocyte clusters (Figure 2). Supplemental Table 1 (supplemental material available online with this article; https://doi.org/10.1172/jci.insight.171984DS1) summarizes the cluster identification and top genes used to identify the unique clusters found within the broader categories. Both broad and subtype categories were utilized to perform subsequent differential expression and communication analyses.

**Figure 3:**
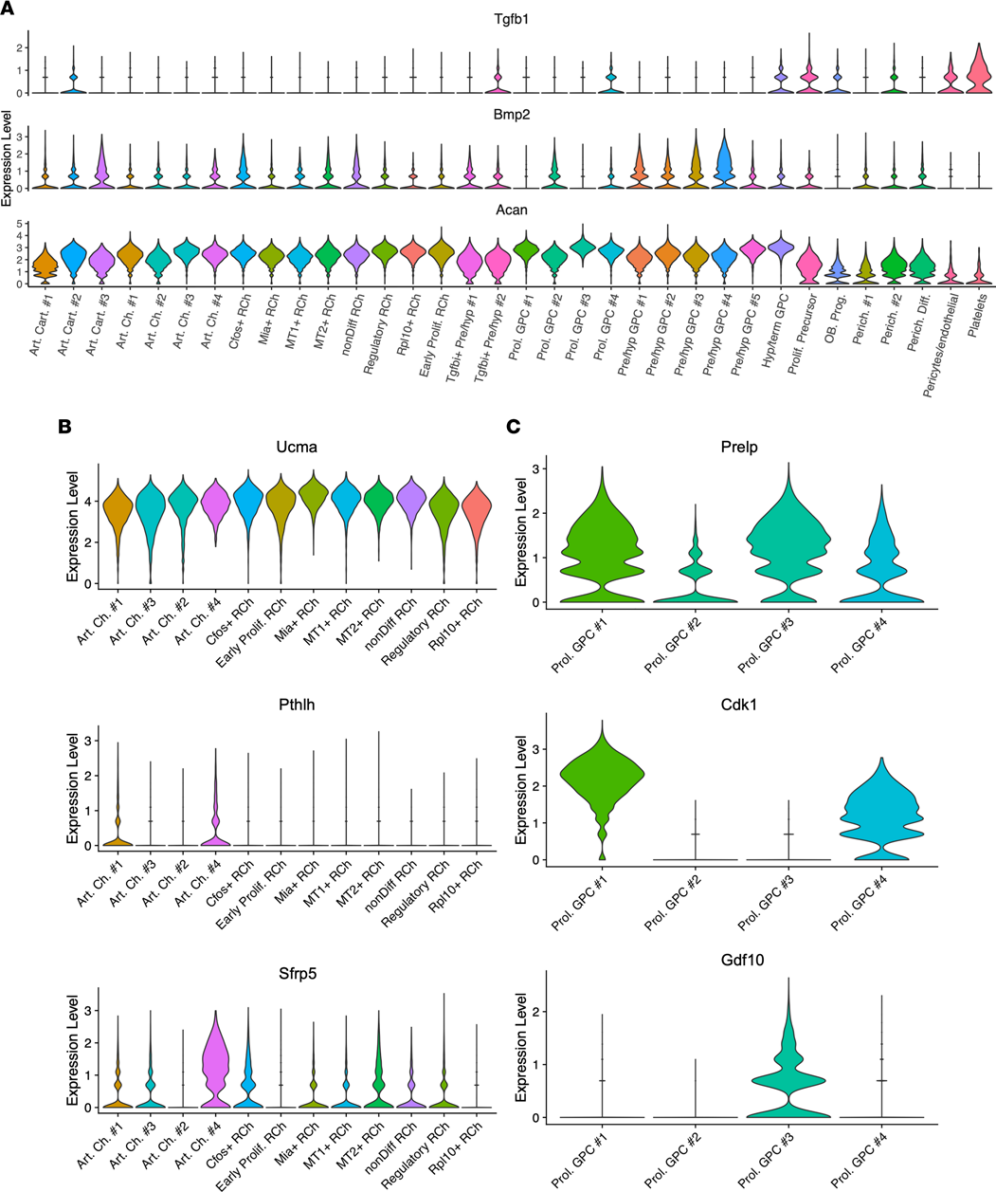
ScRNA-Seq analysis reveals extensive heterogeneity within cell types. (**A**) Violin plots showing the expression of common chondrocyte markers in growth plate tissues. (**B**) Violin plots showing heterogeneity in expression of common resting chondrocyte markers. (**C**) Violin plots showing heterogeneity in expression of common proliferative chondrocyte markers.

Following this, we focused on additional common chondrocyte markers and generated violin plots for *Tgfβ1*, *Bmp2*, *Acan*, *Cdk1*, and *Pthlh*, among others (Figure 3A). We found that markers regularly used to label entire zones of the growth plate were only expressed by some cell clusters within the same category while others expressed different chondrocyte gene hallmarks. For example, whereas all resting chondrocyte clusters expressed *Ucma*, a commonly used marker specific to this cell type, only some clusters expressed *Pthlh* while others expressed *Sfrp5*, both general markers previously used to overall identify resting chondrocytes (Figure 3B) (53–57). In proliferative chondrocytes, *Cdk1* was expressed by clusters 1 and 4, *Gdf10* was expressed by cluster 3, and all clusters expressed *Prelp*, all markers used to generally identify proliferative chondrocytes (Figure 3C) (54, 58). This analysis revealed the unappreciated heterogeneity of chondrocytes present in the growth plate.

We identified 12 clusters of resting chondrocyte cells with distinct expression profiles (Supplemental Figure 1). The clusters were labeled based on either their top distinguishing gene expression marker or the known roles for several top marker genes. For example, some clusters were categorized as articular chondrocytes as the expression of top genes localized them to the resting zone but closer to the articular surface based on markers previously identified with in situ hybridization or laser capture dissection (59, 60). Within the broad differentiating chondrocyte category as defined by *Ihh*, *Cdk1*, and *Runx2* expression among others, we identified 12 distinct clusters with markers representing proliferative (*Cdk1*), prehypertrophic (*Ihh*), and hypertrophic (*Col10a1*) growth plate chondrocytes (Supplemental Figure 2). Based on findings, we captured pre- and early hypertrophic cells, as well as terminally differentiating hypertrophic chondrocytes based on *Col10a1* expression.

In the cells identified as perichondrial, we identified 5 unique clusters that likely represent different types or stages of progenitor cells (Supplemental Figure 3). All shared general markers of perichondrial tissues (*Prrx1*, *Thbs4*, *Dkk3*, *Postn*) as well as high expression of type I collagen. One cluster had high expression of *Ctsk*, suggesting this cluster contained osteoblast progenitors. Perichondrial clusters 1 and 2 expressed multiple perichondral markers, including *Col1a1*, *Col3a1*, and *Periostin*, but also unique markers. For instance, perichondrial 1 showed high expression of *Prrx1*, *Tgfbi*, *Hes1*, and *Axin2*, and perichondrial 2 showed high expression of *Tnn* and *Ltbp2* but lower expression of *Prrx1*. The cluster designated as proliferative chondrogenic precursor expressed all the perichondrial markers, as well as high levels of *Cdk1*, an indication of progenitor cells with more proliferative potential, likely in the process of early differentiation. The perichondrial diff. cluster highly expressed *Col2a1* but with lower expression of other perichondrial markers, indicating the cells are in an advanced state of differentiation. There were also 3 unique articular cartilage clusters expressing high or mid-levels of *Prg4*, and previous work showed that *Prg4*-expressing cells in embryonic joint constitute a progenitor pool for all regions of articular cartilage in the adult (Figure 2D) (61). A cluster represented by *Notch3* and *Cdh5* expression likely represented pericyte and endothelial cells associated with vascularization necessary for perichondrium function (Figure 2D) (62, 63). This cluster analysis showed the complexity of the captured cartilage cell types and that noncartilaginous tissues including bone, tendon, ligament, and muscle were avoided. We next sought to determine how gene expression in these cartilage cell types and cluster subtypes was affected in the *Aga2^+/–^* mouse.

### Type I collagen is expressed in perichondrial cells and developing chondrocytes.

**Figure 4:**
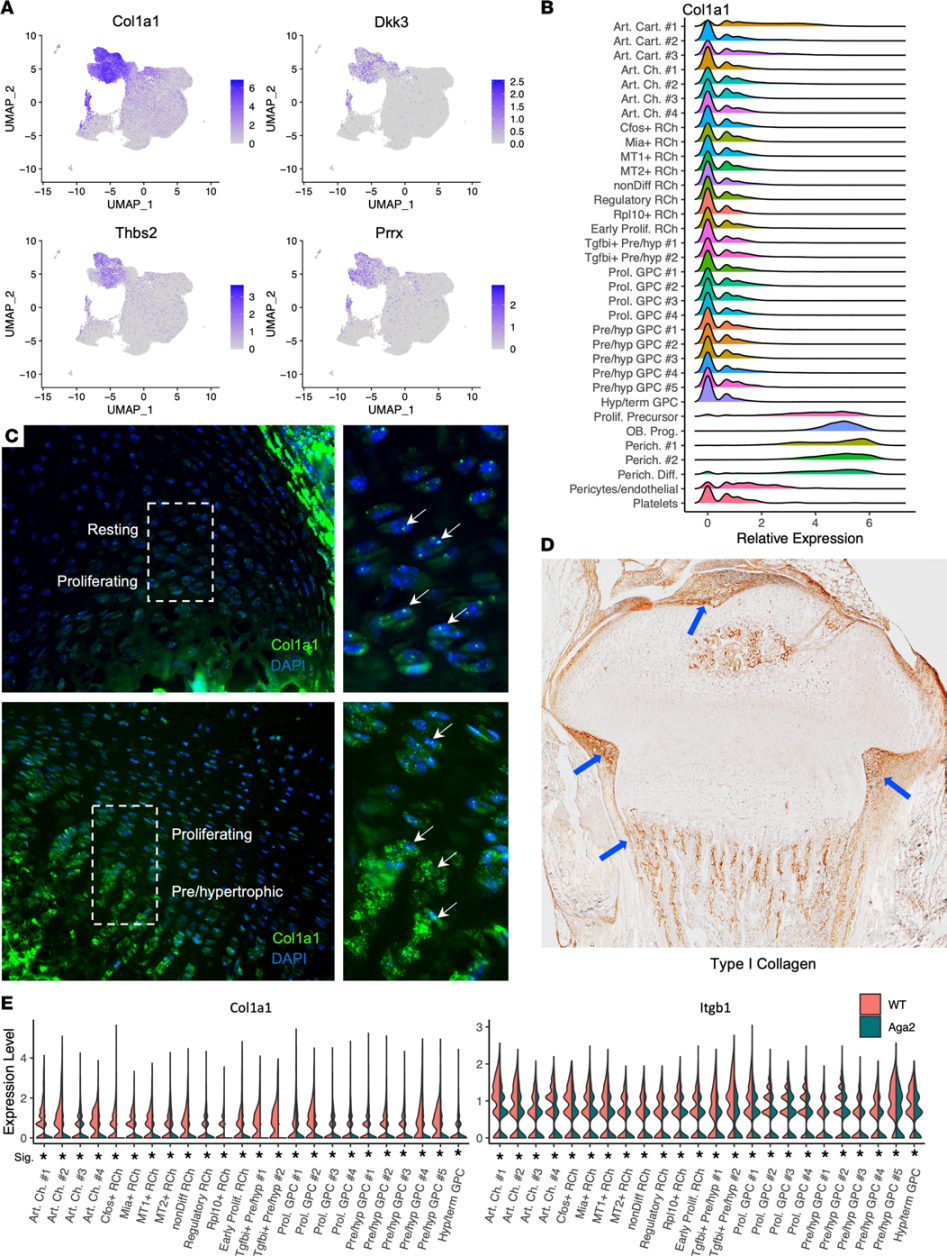
Type I collagen is expressed in the perichondrium and the growth plate. (**A**) Feature plots showing the coexpression of Col1a1 and multiple perichondrial markers. (**B**) Ridge plot showing high expression of Col1a1 in perichondrial clusters but also low expression in cartilage clusters. (**C**) RNAscope for Col1a1 (green) costained with DAPI (blue) indicating Col1a1 expression in resting, proliferating, and pre/hypertrophic cells in a P8 WT growth plate. White arrows point to RNAscope signal. *N* = 3. (**D**) Immunohistochemistry using an antibody against type I procollagen in a P8 WT growth plate. Blue arrows point to type I procollagen protein signal. *N* = 3. (**E**) Violin plots showing increased Col1a1 and Itgb1 expression in growth plate cell clusters. * = *P* < 0.05.

Since the *Aga2^+/–^* mouse harbors a *Col1a1* mutation, we determined where type I collagen (encoded by *Col1a1* and *Col1a2*) RNA was expressed. Our scRNA-Seq data showed that *Col1a1* and *Col1a2* were highly expressed in perichondrium clusters (coexpressed with known perichondrial marker genes *Thbs2* and *Dkk3*) (Figure 4A) (31). There were unexpectedly low levels of *Col1a1* expression in other growth plate zones, particularly resting and differentiating chondrocytes (Figure 4B). To verify this, we performed in situ hybridization for *Col1a1* expression in P5 mouse growth plates using the RNAscope platform (ACDBio) and demonstrated low expression of *Col1a1* throughout chondrocyte cells with highest levels in differentiating chondrocytes (Figure 4C). This was not observed at the protein level, where immunohistologic analyses of WT (P8) mouse growth plates showed expression of type I collagen in cells lining the articular surface chondrocytes, late hypertrophic chondrocytes, the perichondrium, and the groove of Ranvier (Figure 4D). These findings from the single-cell data and verified by RNAscope support that type I collagen was expressed at low RNA levels throughout growth plate tissues, and its expression, in both WT and *Aga2^+/–^* mice, may affect growth plate dynamics. Comparing *Col1a1* expression between WT and *Aga2^+/–^* cells showed markedly decreased *Col1a1* expression in nearly all *Aga2^+/–^* chondrocyte clusters, which falls in line with previous reports of reduced *Col1a1* RNA expression in the *Aga2^+/–^* mouse (64). The expression of *Itgb1*, a known Col1a1 binding partner, was also significantly decreased in all *Aga2^+/–^* cells, independently supporting the scRNA-Seq findings (Figure 4E) (65).

### The Aga2^+/–^ mutation contributes to increased UPR via the PERK pathway in differentiating chondrocytes and perichondrial tissues.

**Figure 5:**
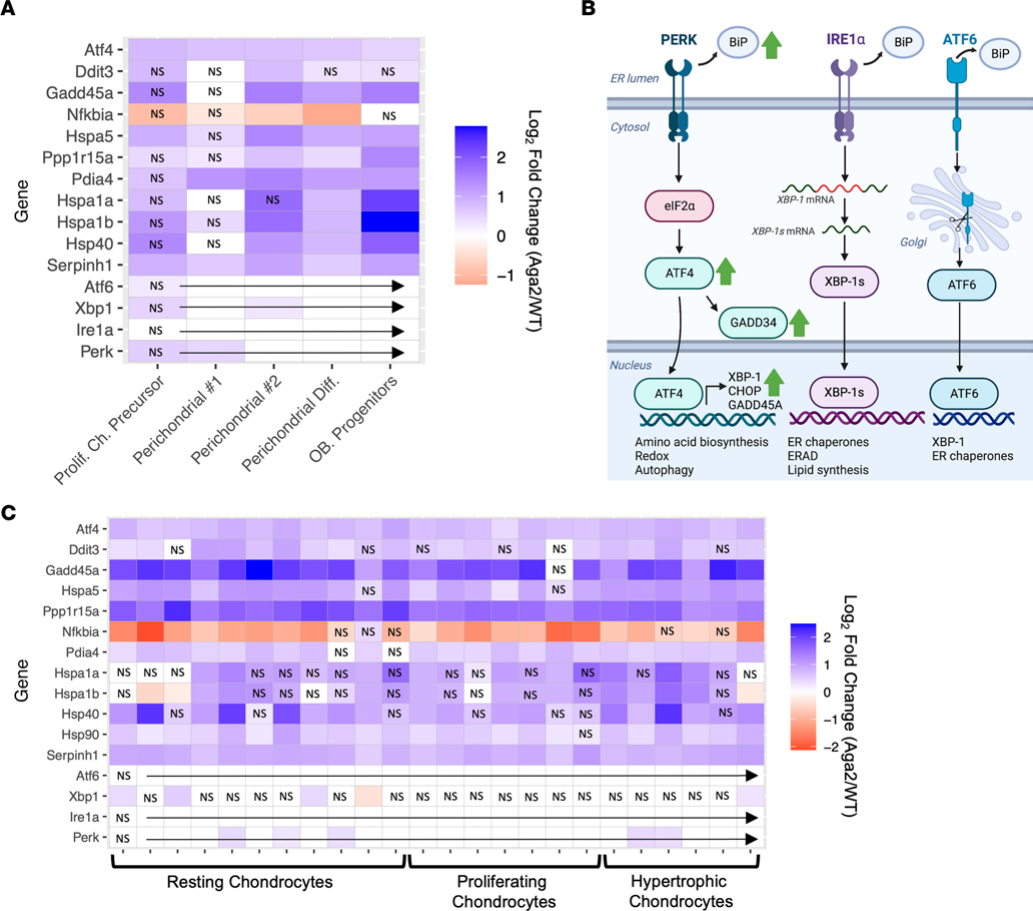
ER stress marker expression is increased in both *Aga2^+/–^* perichondrial and chondrocyte cell populations. (**A**) Heatmap showing differential expression of UPR signaling pathway components as indicators of ER stress in perichondrial clusters. (**B**) Illustration showing the UPR pathways, highlighting that increased expression was mainly seen in the PERK pathway (green arrows). ERAD, ER-associated protein degradation; XBP-1, X-box binding protein 1. (**C**) Heatmap showing differential expression of UPR signaling pathway components as indicators of ER stress in resting and differentiating chondrocyte clusters. NS spaces represent differential expression values that were not significant.

The *Aga2^+/–^* mouse is an established model to study ER stress in type I collagen–expressing tissues. However, whereas ER stress has been previously observed in OI hypertrophic chondrocytes in the *Col1a2^+/p.G610C^* mouse, little is known about ER stress in *Aga2^+/–^* perichondrial/periosteal or other growth plate chondrocyte cells (19). Perichondrial/periosteal cells express *Prrx1* (a common marker for osteochondroprogenitors) and have the highest level of type I collagen expression in growth plates as well as the ability to differentiate into both chondrocytes and osteoblasts (66–68). *Aga2^+/–^* clusters with high *Prrx1* expression (identified in Figure 2D) showed significantly increased levels of several established ER stress markers (*Gadd45a*, *Hspa5* *[Bip]*, *Serpinh1* *[Hsp47]*, *Atf4*, *Ddit3* *[Chop]*, *Ppp1r15a* [*Gadd34*]; ref. 69) as well as decreased expression of *Nfkbia*, a gene known to be negatively regulated by the PERK pathway (Figure 5A) (70, 71). These markers are known to be downstream of PERK activation in the UPR and fall in line with earlier work showing increased PERK activation in *Aga2^+/–^* osteoblast cultures and OI patient fibroblasts (41, 42, 46). Other UPR receptors (*Atf6* and *Ire1a*) and their downstream effectors, however, are not differentially expressed in *Aga2^+/–^* perichondrial clusters, supporting the idea that ER stress activation in OI mesenchyme-derived tissues appears to favor the PERK pathway of the UPR (Figure 5B). Additionally, *Aga2^+/–^* growth plate chondrocytes similarly showed upregulation of ER stress markers, including *Gadd45a*, *Hspa5* *[Bip]*, *Serpinh1* *[Hsp47]*, *Atf4*, *Ddit3* *[Chop]*, and *Ppp1r15a* (*Gadd34*) (Figure 5C). These increases in ER stress activation likely have an impact on cell function and signaling. Although ER stress has been shown to be overactivated in the *Aga2^+/–^* mouse bone due to type I collagen retention in the ER, MAPK signaling also activates the UPR through the activation of CHOP by P38 (72). As delineated below, there was increased MAPK signaling through elevated FGF signaling in *Aga2^+/–^* growth plate chondrocytes, and this activation may contribute to the ER stress response concurrently with the cell’s response to mutant type I collagen.

### Chondrocyte differentiation pathways are altered in Aga2^+/–^ cartilage, leading to increased differentiation.

**Figure 6:**
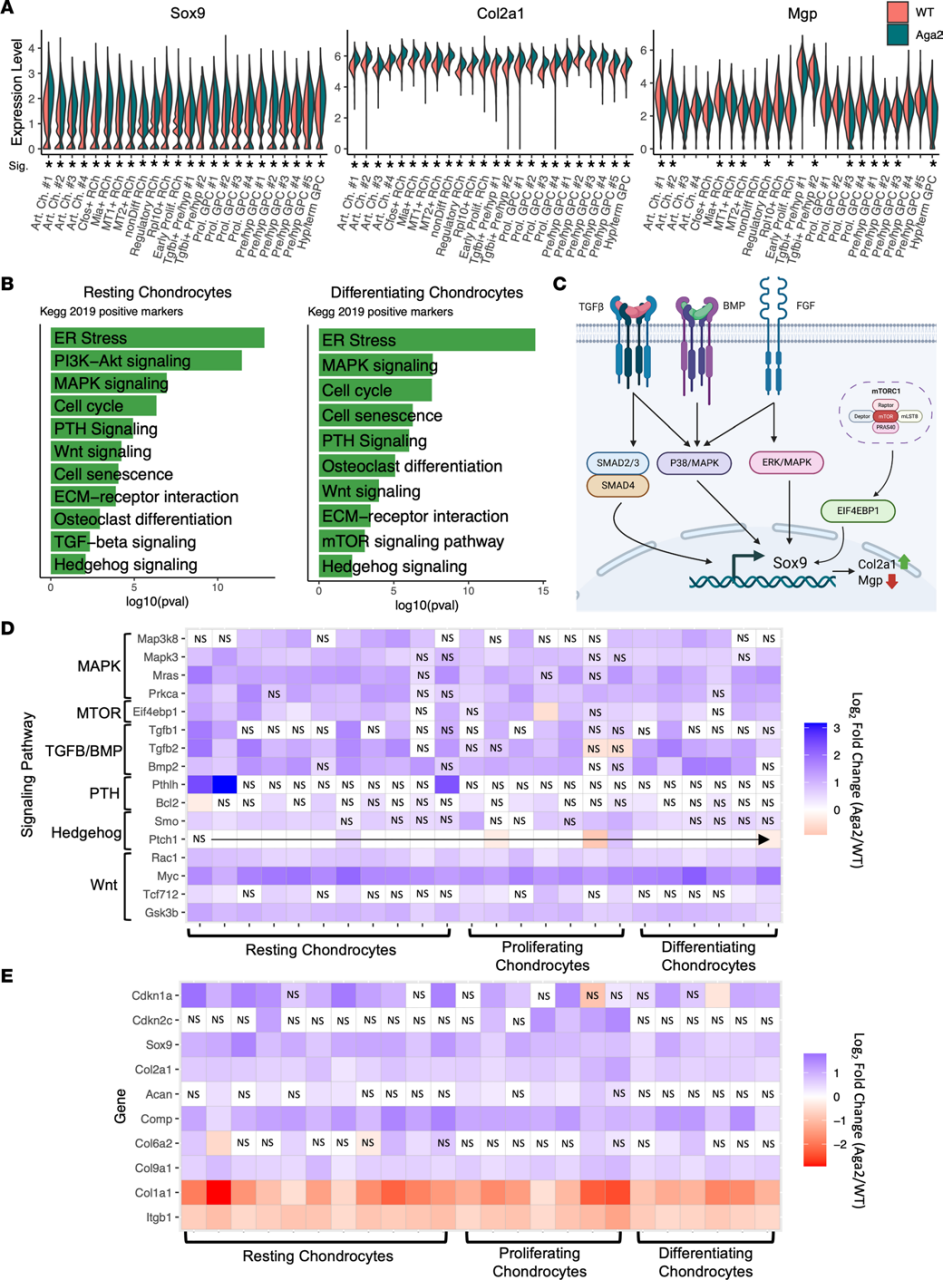
Resting and differentiating *Aga2^+/–^* chondrocyte differential expression indicates accelerated differentiation. (**A**) Violin plots showing increased Sox9 and Col2a1 as well as decreased Mgp expression in resting and differentiating chondrocytes. * = *P* < 0.05. (**B**) Top relevant enriched KEGG pathway analysis terms between WT and *Aga2^+/–^* in resting and differentiating chondrocytes. (**C**) Illustration showing the multiple pathways influencing Sox9 expression. (**D**) Heatmap showing differential expression of signaling pathway components important in chondrocyte differentiation. (**E**) Heatmap showing differential expression of additional cell cycle and cartilage markers. NS spaces represent differential expression values that were not significant.

Proper endochondral bone formation requires a delicate balance of signaling pathways and transcription factor activity. A master transcription factor, *Sox9*, is required to maintain chondrocyte identity, while preparing differentiation into hypertrophy. Under normal conditions, decreasing levels of *Sox9* expression contribute to chondrocytes transitioning to terminal hypertrophy and ultimately osteoblasts (50). However, continued expression of *Sox9* throughout cells derived from the chondrocyte lineage inhibits terminal differentiation of hypertrophic chondrocytes as well as their transition into osteoblasts (73). Our single-cell analysis found significant upregulation of *Sox9* as well as its main nuclear target, *Col2a1*, throughout all growth plate clusters (Figure 6A). *Mgp*, a marker for terminal growth plate chondrocytes, was recently shown to be negatively regulated by SOX9, with *Sox9* inactivation resulting in increased *Mgp* expression and increased terminal chondrocyte differentiation (50, 74). We found significant downregulation of *Mgp* in many growth plate clusters, including *Aga2^+/–^* hypertrophic/terminal chondrocytes, possibly indicating inhibition of terminal chondrocyte differentiation (Figure 6A). Persistently elevated *Sox9* expression induces proliferating chondrocytes to undergo accelerated hypertrophy, likely contributing to the shorter proliferative zone observed in vivo in the *Aga2^+/–^* mouse.

In addition to *Sox9* acting as a master driver of chondrocyte differentiation, there are several signaling pathways that also regulate this process. For example, proliferating chondrocytes exit the cell cycle and enter hypertrophy, through hedgehog signaling and the upregulation of *Ihh* and *Pth* expression (75). Interactions between IHH, PTH-related peptide (PTHLH), BMP, WNT, and FGF signaling pathways balance chondrocyte proliferation versus differentiation, influencing growth plate development and longitudinal bone growth. To determine the effects of a type I collagen mutation on growth plate development dynamics, we performed Kyoto Encyclopedia of Genes and Genomes (KEGG) pathway analysis on resting and differentiating chondrocytes (Figure 6B). MAPK signaling activation induces chondrocyte proliferation and promotes differentiation and was identified as one of the top upregulated processes in the KEGG pathway analysis, with increased expression of 29 pathway genes (Supplemental Table 1) (76). As an example, *Map3k8*, an activator of MAPK and JNK signaling, was upregulated in several resting and differentiating chondrocyte clusters and supports the upregulation and pathogenic role of MAPK signaling in *Aga2^+/–^* chondrocytes (77). As noted above, *Sox9* expression was increased, and it is induced by several signaling pathways (Figure 6C). MAPK signaling is a potent inducer of *Sox9* expression in the cartilage growth plate specifically when activated by FGF through MAPK/ERK signaling (78). In further support of the MAPK-Sox9 connection, the analysis showed increased *Ras* and *Prkca* expression, transcription factors involved in the MAPK/ERK pathway (Figure 6D). MTOR signaling has also been shown to induce *Sox9* expression and was also identified as an upregulated pathway in KEGG analysis. A recent study revealed that MTORC1 controls *Sox9* expression through activation of EIF4EBP1 or -2, and our analysis showed significantly increased *Eif4ebp1* expression in resting, proliferative, terminal hypertrophic, and perichondrial clusters (Figure 6D) (79). TGF-β/BMP signaling is also an inducer of *Sox9* expression, and both *Tgfb2* and *Bmp2* expression were increased in several subtypes of resting cells as well as proliferative and prehypertrophic cell clusters (80). We identified increased Sox9 expression throughout growth plate clusters and showed established pathways that regulate Sox9 expression, including MAPK, MTOR, and TGF-β/BMP, were also elevated.

Additional signaling pathways were also dysregulated in *Aga2^+/–^* growth plate chondrocytes. In both resting and differentiating cells there was upregulation of PTH and WNT signaling pathways along with cell cycle genes and ECM receptor interactions (Figure 6, D and E). The KEGG analysis findings also suggested increased evidence of differentiation. *Tgfβ2* and *Bmp2* are mainly expressed in the hypertrophic zone and are essential for the induction of chondrocyte differentiation (81, 82). In *Aga2^+/–^* chondrocytes, *Tgfβ2* and *Bmp2* expression increased in resting, proliferative, and prehypertrophic cell clusters, suggesting that there is potential induction of chondrocyte differentiation. PTH signaling in the growth plate is an inducer of proliferation in columnar chondrocytes and a suppressor of their differentiation into postmitotic hypertrophy (83, 84). In *Aga2^+/–^*, there was increased expression of *Pthlh* as well as its downstream target *Bcl2* in multiple articular and resting chondrocyte clusters, suggesting accelerated proliferation from resting to proliferative cells (84). Canonical WNT signaling is an inhibitor of chondrogenesis and stimulator of chondrocyte hypertrophy (85). In both resting and differentiating chondrocytes, the WNT signaling effector *Gsk3b* was increased along with *Rac1* and *Myc*, both nuclear WNT targets (Figure 6D) (85). Both *Rac1* and *Myc* have been shown to play roles in inducing chondrocyte proliferation and differentiation (86, 87). Interestingly, MYC has also been shown to impair terminal hypertrophy and calcification in chondrocytes, and our data also reveal increased *Myc* expression in hypertrophic/terminal chondrocytes (Figure 6D) (88). This indicates a possible inhibition of terminal differentiation in *Aga2^+/–^* chondrocytes, which would affect the primary spongiosum and bone. The canonical WNT signaling target *Tcf7l2* expression is also upregulated in Rpl10^+^ resting chondrocytes and a proliferative chondrocyte cluster, overall indicating an increase in canonical WNT signaling activation, which is predicted to accelerate chondrocyte differentiation (Figure 6D).

Evaluation of proliferation gene expression markers found increased expression of *Cdkn1a* and *Cdkn2c*, both cell cycle inhibitors, in a number of resting and differentiating chondrocyte subsets, and importantly in proliferative chondrocytes (Figure 6E). *Cdkn1a* encodes for the P21 protein, and its increased expression results in inhibition of chondrocyte growth (89). The expression of some ECM proteins was also increased, such as type II collagen, type IX collagen, aggrecan, and cartilage oligomeric matrix protein, while *Col1a1* and its primary integrin binding partner, *Itgb1*, showed decreased expression (Figure 6E). The upregulation of TGF-β/BMP and WNT signaling genes; an increase in *Cdkn1a* expression in resting, proliferating, and differentiating chondrocytes; and the histologic finding of a shortened proliferative zone suggests that the *Aga2^+/–^* chondrocytes are affected by decreased proliferation and accelerated differentiation.

### Aga2^+/–^ perichondrial progenitor cells show altered cell cycle activation and differentiation.

**Figure 7:**
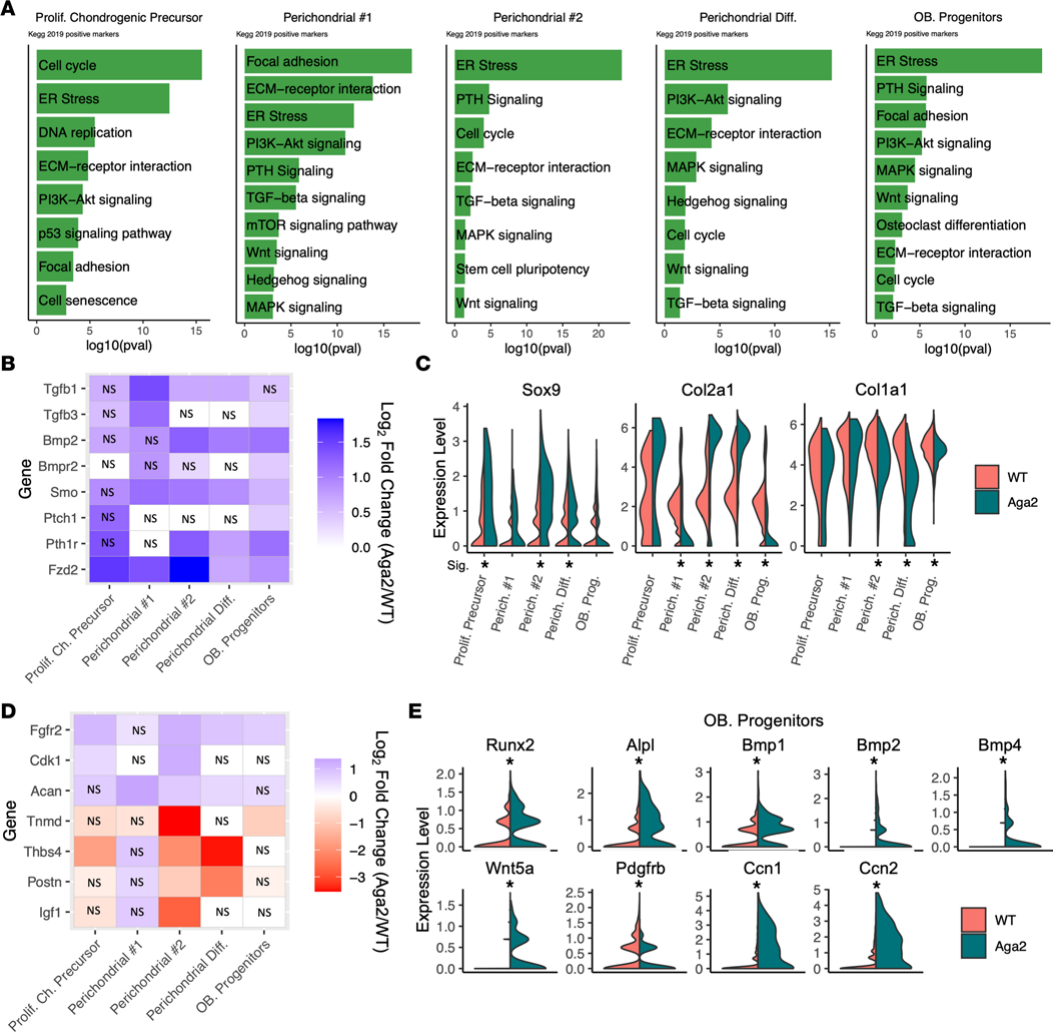
*Aga2^+/–^* perichondrial cell differential expression indicates altered cell cycle and accelerated differentiation. (**A**) Top relevant enriched KEGG pathway analysis terms between WT and *Aga2^+/–^* in individual perichondrial cell clusters. (**B**) Heatmap showing differential expression of signaling pathway components important in chondro-progenitor differentiation. (**C**) Violin plots showing increased Sox9 and Col2a1 as well as decreased Col1a1 expression in perichondrial cell clusters. (**D**) Heatmap showing differential expression of additional cell differentiation and perichondrial markers. NS spaces represent differential expression values that were not significant. (**E**) Violin plots showing increased osteoblast differentiation markers as well as decreased Pdgfrb expression in the osteoblast progenitor cluster. * = *P* < 0.05.

The perichondrium and groove of Ranvier contain cartilage progenitor cells, important for appositional cartilage growth, and serve as signaling centers that communicate with the growth plate (26, 31). In cells identified among the perichondrial clusters, KEGG pathway analysis was performed on the differential expression between WT and *Aga2^+/–^* samples. The analysis revealed enrichment of TGF-β, PTH, WNT, and MAPK signaling pathways as well as cell cycle, focal adhesion, and ECM receptor interactions (Figure 7A). In comparison with growth plate chondrocytes, perichondrial cell clusters expressed significantly higher amounts of type I collagen and, in *Aga2^+/–^*, increased amounts of mutant type I collagen. This led to abnormal increases in signaling pathway activation, similar pathways seen in chondrocyte clusters, but at a more pronounced level. *Bmp* and *Tgfβ* ligands are normally expressed in the perichondrium. Among the perichondrial clusters, *Tgfβ1* had increased expression particularly in perichondrial 1 and differentiating perichondrial clusters, and we saw elevated expression of *Tgfβ3* in the osteoblast progenitor and perichondrial 1 clusters. Increases in TGF-β signaling promoted osteoprogenitor proliferation and early differentiation (Figure 7B) (90). There was also upregulation of *Bmp2* and *Bmpr2* in multiple perichondrial clusters, and increased BMP signaling in the perichondrium leads to accelerated progenitor differentiation (91). Among the other pathways identified as altered were upregulated *Pth1r* in multiple clusters and *Fzd2*, a receptor involved in WNT signaling, both of which play important roles in chondrocyte differentiation (Figure 7B) (85, 92).

Similar to chondrocytes, there were alterations in cell cycle and differentiation markers within *Aga2^+/–^* perichondrial clusters. Perichondrial 2 and perichondrial differentiating clusters showed increased expression of *Sox9* and *Col2a1*, a known target of SOX9. Decreased *Col1a1* expression was also observed in *Aga2^+/–^* progenitor cells, consistent with previous studies in *Aga2^+/–^* tissues showing decreased *Col1a1* expression (Figure 7C) (64). Changes in expression of additional progenitor differentiation markers in perichondrial clusters (*Fgfr2*, *Acan*, *Tnmd*, *Thbs4*, *Postn*, *Igf1*, *Cdk1*) indicated alterations in progenitor cell differentiation and proliferation (Figure 7D) (26, 31, 93–98). The cluster identified as osteoblast progenitors showed increased *Runx2* expression along with other osteoblast differentiation markers, including *Alpl*, *Bmp1*, *Bmp2*, *Bmp4*, and *Wnt5a*, further suggesting accelerated differentiation in these perichondrial cells (Figure 7E). We also saw a decrease in *Pdgfrb*, an established osteoblast progenitor marker (Figure 7E) (99). PDGFR signaling has been shown to negatively regulate osteogenesis, further supporting the enhanced differentiation of *Aga2^+/–^* perichondrial cells. *Aga2^+/–^* osteoblast progenitors also showed increased levels of *Ccn1* and *Ccn2* expression (Figure 7E) (100). Both *Ccn1* and *Ccn2* play important roles in osteoblastogenesis, with *Ccn2* particularly influential in osteoblast differentiation and increased *Alpl* expression and activity (101). The data suggest an acceleration in early osteoblast differentiation in *Aga2^+/–^* perichondrial derived cells.

Genes associated with cell cycle were the top upregulated KEGG term in the perichondrial cluster designated as proliferative chondrogenic precursors. These results indicate that with increased cell cycle activation, proliferative precursors are likely undergoing a reduction in differentiation. However, those progenitor cells that do manage to undergo early differentiation seem to differentiate more quickly. Both effects could markedly reduce the pool of progenitor cell populations that contribute to the growth plate. These data suggest alterations in progenitor cell function and differentiation potentially lead to a reduced progenitor pool necessary for endochondral ossification and osteoblast maturation.

### FGF signaling expression and communication are upregulated in perichondrial and growth plate tissues.

**Figure 8:**
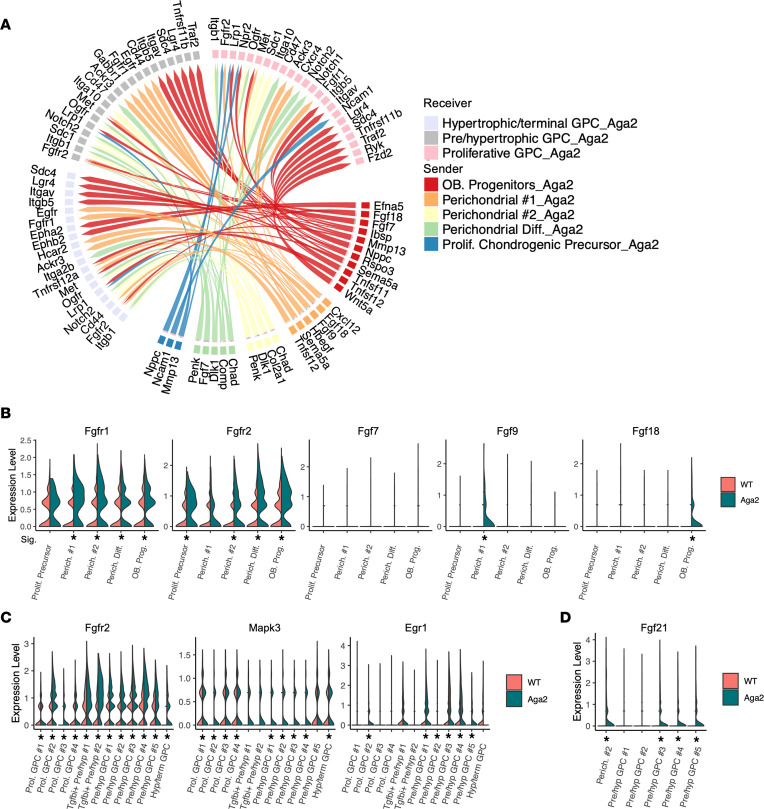
Altered cell communication between progenitors and growth plate cells in *Aga2^+/–^* cartilage. (**A**) Circos plot showing inferred upregulated cell communication via NicheNet analysis. Differentiating receiver chondrocytes with increased expression of signaling receptors and downstream targets are connected to the perichondrial sender cell types expressing ligands predicted to promote this response. Ligands expressed by the same cell population are colored the same. (**B**) Violin plots showing expression of FGF receptors and ligands in perichondrial clusters. (**C**) Violin plots showing increased FGFR2 receptor, downstream signal transducer MAPK, and nuclear target EGR1 expression in differentiating chondrocytes. (**D**) Violin plot showing increased FGF21 in perichondrial 2 cluster and multiple pre/hypertrophic chondrocyte clusters. * = *P* < 0.05.

Signaling communication between the perichondrium and cartilage growth plate is essential for proper endochondral bone growth. To address the role of tissue communication, we performed analysis of our single-cell data using the NicheNet package, which determines cell communication by using the expression data of cells known to interact and combines them with existing knowledge of ligand-receptor-target interactions (101). We designated our perichondrial cells as senders and differentiating chondrocytes as receivers (Figure 8A). We saw alterations in several pathways, including upregulation of Wnt and Notch signaling communication. Interestingly, although we saw increases in TGF-β pathway gene expression within clusters, our NicheNet analysis revealed decreased TGF-β as well as Igf signaling communication between the perichondrium and growth plate (Supplemental Figure 4). Most consistently, however, the analysis showed upregulation of FGF signaling communication specifically from perichondrial cells 1 (*Fgf9* and *Fgf18*) and osteoblast progenitors (*Fgf7* and *Fgf18*).

Further analyses were performed based on the rationale that activating mutations in *FGFR1* produce the condition osteoglophonic dysplasia, which is characterized by low bone mineral density, nonossifying bone lesions, and fracture-healing deficits, findings seen in OI (102). FGFR1 is highly expressed in the perichondrium, and it is coupled to skeletal homeostasis through osteoprogenitor populations (34). Additionally, FGF signaling is important in perichondrium/growth plate communication. In *Aga2^+/–^* perichondrial clusters there was upregulation of *Fgfr1* and *Fgfr2* expression. Correlatively, there was increased expression of FGF ligands including *Fgf9* and *Fgf18* in specific perichondrial clusters (Figure 8B). Though *Fgf7* is highly expressed in both cartilage and the perichondrium, and while its definitive role remains unclear, exogenous FGF7 facilitates osteogenic differentiation in mouse embryonic fibroblasts (103). Transgenic mice overexpressing *Fgf9* show short limbs due to reduced chondrocyte proliferation similar to bone phenotypes caused by activated FGFR3 as seen in achondroplasia (104). Stimulation of chondrogenic cultures with FGF18 accelerates chondrocyte differentiation and FGF18-treated cultured fetal mouse tibia show decreased bone length (105, 106). We also observed significantly increased *Fgfr2* expression throughout resting and differentiation chondrocyte clusters (Figure 8C). In agreement, there was upregulation of FGF signaling effectors *Mapk3/Mapk1* and the nuclear target *Egr1* (Figure 8C) (107). Additionally, we observed increased *Fgf21* expression in perichondrial 2 and multiple prehypertrophic chondrocyte clusters (Figure 8D). The ligand FGF21 is associated with ER stress in a mouse model of metaphyseal chondrodysplasia Schmid type and shown to be induced by ATF4 and CHOP nuclear binding (108–110). We verified *Fgf9*, *Fgf18*, and *Fgf21* spatial expression in the WT perichondrium and specific growth plate zones using RNAscope (Supplemental Figure 5), validating our scRNA-Seq clustering data. Overall, these findings present FGF signaling as a possible target in OI that may affect growth plate dynamics and progenitor development.

## Discussion

Short stature is a phenotypic finding in all types of OI, and the effect of type I collagen on growth plate dynamics is an understudied subject. In this study, we showed growth plate abnormalities in the *Aga2^+/–^* mouse, an OI model harboring a dominant mutation in type I collagen. We showed marked decreases in proliferative zone length and no changes in the hypertrophic zone length. This differs from the findings observed in Scheiber et al. of decreased overall bone length but increased overall growth plate height in the G610C mouse model, an OI model harboring a missense mutation in *Col1a1* (19). Additionally, the measurements, made at much later time points than in our study, could reflect differences between early and later postnatal endochondral growth as well as differing genotypes.

In our scRNA-Seq analysis on WT cartilage, we found that resting and differentiating chondrocytes as well as perichondrial cells showed marked levels of cell heterogeneity. This heterogeneity was identified not only by the top markers generated during unsupervised cluster analysis but also by using markers from the literature. While genes like *Pthlh* are reported to be expressed throughout the resting zone, we found that only some resting clusters expressed *Pthlh* while others expressed different markers such as *Ucma* or *Sfrp5*. This heterogeneity extended to all growth plate chondrocyte cells or historically established zones. The perichondrium also showed heterogeneity with some clusters expressing *Prrx1* while others expressed *Hes*1 as a top marker. Yet all clusters expressed high amounts of *Col1a1*, supporting the immunohistochemistry that type I collagen is expressed throughout the perichondrium. Other cartilage scRNA-Seq studies have shown similar levels of heterogeneity in cartilage tissues but did not use it as a focus in their analyses (48–51). Our reporting of this cell heterogeneity illustrates the advantage that scRNA-Seq analysis has in investigating complex growth plate dynamics while allowing for analyses of changes in lowly expressed genes under pathologic conditions when compared with protein analyses.

When we performed differential expression analysis in WT versus *Aga2^+/–^*, we observed increased expression of several markers of chondrocyte differentiation as well as decreased proliferation correlated with increased *Cdkn1a* expression (89). This would contribute to the shorter proliferative zone observed in vivo and disrupted overall long bone growth, which is seen in the *Aga2^+/–^*. In addition to accelerated differentiation in *Aga2^+/–^* chondrocytes, there was also diminished differentiation of terminal chondrocytes, critical to endochondral ossification’s transition to bone. A recent study showed that terminal hypertrophic chondrocytes in the G610C OI mouse model stagnate in the growth plate, inhibiting their differentiation into osteoblasts (19). Supporting decreased terminal chondrocyte differentiation, we showed increased *Myc* and decreased *Mgp* expression in terminal hypertrophic chondrocytes. This increase in differentiation, but delayed terminal hypertrophy, could be due to the increased *Sox9* expression observed throughout the growth plate. In early chondrocyte maturation, SOX9 is required to maintain the chondrocyte phenotype while also inducing chondrocyte exit from the proliferative cycle and into prehypertrophy (111). At terminal differentiation, however, *Sox9* levels normally decrease, but in *Aga2^+/–^* hypertrophic cells, *Sox9* levels continue to be maintained. Persistent *Sox9* expression in hypertrophic cells predicts a delay in terminal differentiation as previously demonstrated (50). Therefore, inappropriate *Sox9* expression could be one of the driving elements preventing terminal chondrocyte maturation and thereby affecting the coordinated process of endochondral ossification. *Sox9* expression is induced by many signaling pathways, and, in our analysis, we observed the dysregulation of BMP, MAPK, and MTOR signaling pathways throughout the growth plate, known inducers of *Sox9* expression (111).

In the analysis of WT growth plate cells, we found high expression of *Col1a1* in the perichondrium, as expected, but low expression was also observed in resting and differentiating chondrocytes, cells that are normally known to express high levels of type II but not type I collagen. This was confirmed via RNAscope in situ hybridization and has not been previously appreciated. These data raise the questions, first, what is the role for type I collagen gene expression in chondrocytes, and second, how then does a *Col1a1* mutation cause alterations in growth plate chondrocyte mechanics? The *Aga2^+/–^* mouse is an established OI model to study the effects of ER stress in bone, and *Aga2^+/–^* affected osteoblasts showed dilated ER, retained procollagen molecules, and increased apoptosis (18). Elaborate ER quality control systems ensure efficient synthesis and prevent aggregation of proteins. This includes the UPR, which is activated upon accumulation of misfolded proteins beyond cellular capacity. The UPR is an adaptive ER response involving upregulation of several stress-responsive markers including BIP, HSP47, CHOP, and ATF4 (reviewed in ref. 69). The initial stage of the UPR is to restore ER homeostasis to preserve cell function, the homeostatic UPR (112). If the capacity of the ER to fold proteins or its quality control machinery is overwhelmed, accumulation of misfolded proteins leads to “ER stress,” altering cell function and survival. Numerous studies demonstrate that ER stress occurs in OI (18, 19, 42, 44). Biallelic mutations in the genes encoding the type I procollagen chaperones, *CRTAP*, *PPIB*, *LEPRE1*, *LH2*, *FKBP65*, and *HSP47*, and dominant type I procollagen gene mutations have all been shown to lead to ER stress and cellular dysfunction (19, 41, 43–45). Varying degrees of ER stress have also been demonstrated in multiple mouse models (18, 19, 43). We and others have also shown that treatment for ER stress via 4PBA resulted in increased mouse linear growth (46, 113). This study found increased markers of ER stress throughout the growth plate, particularly the effectors *ATF4* and *CHOP*. These effectors are downstream of the PERK arm of the UPR pathway. This confirms our and others’ previous findings that UPR activation occurs mainly through the PERK pathway in the context of type I collagen mutations (41, 42, 46). In a model of metaphyseal chondrodysplasia type Schmid, a chondrodysplasia characterized by short stature and ER stress in the growth plate because of *Col10a1* mutations, mice exhibit short stature because of increased expression of *Sox9* via ATF4 and CHOP transactivation (108). Increased *Sox9* expression with accelerated chondrocyte differentiation and delayed terminal hypertrophic differentiation as seen in our *Aga2^+/–^* mice could in part be due increased levels of ER stress markers ATF4 and CHOP stemming from mutant *Col1a1* expression in growth plate chondrocytes in a cell-autonomous fashion as well as driven by TGF-β/BMP, MAPK, and MTOR signaling pathways.

We observed upregulated MAPK signaling throughout the growth plate. MAPK signaling activation is downstream of several receptor types, including receptor tyrosine kinases (RTKs) and integrins. The data showed decreased expression of *Itgb1*, for which type I collagen is a main binding partner, consistent with the findings of decreased type I collagen expression (114). However, *Col2a1* expression was increased (likely because of increased *Sox9* expression), which could activate integrins and therefore MAPK signaling. ER stress has also been shown to induce MAPK activation, demonstrating how multiple cellular signaling mechanisms that are known to crosstalk can perturb cellular function (72, 115, 116). Additionally, MAPK signaling activated by TGF-β and BMP positively regulates *Runx2* expression and function to promote differentiation, expression findings seen in our *Aga2^+/–^* osteoblast progenitor cluster (82). Finally, there was increased *Fgfr2* expression throughout the growth plate, an RTK that signals via MAPK activation. MAPK signaling promotes chondrocyte differentiation and could be contributing to the accelerated differentiation observed in our *Aga2^+/–^* growth plates (76). Therefore, it is unclear how low levels of mutant type I collagen expression in chondrocytes produces the effects of altered signaling activation as a driver behind growth plate abnormalities in OI. Yet our study converges on Sox9 as a critical target in our mouse model.

Perichondrial tissues are known to express high levels of type I collagen, and much of growth plate development depends on the recruitment of progenitor cells and signaling communications from the perichondrium. Several perichondrial clusters showed accelerated differentiation to either the chondrocyte or osteoblast lineage. We also observed dysregulation of ligand expression and signaling activation between perichondrial and chondrocyte tissues via NicheNet analysis, specifically FGF, WNT, and Notch signaling. ER stress markers, identical to those seen in growth plate chondrocytes, were also significantly upregulated in *Aga2^+/–^* perichondrial cells. These alterations could be leading to a depletion in progenitors contributing to the growth plate, thereby affecting linear growth and well as osteoblast progenitors and ultimately fracture healing.

Despite having multiple cellular pathway dysregulations in *Aga2^+/–^* cartilage growth plate, our analysis revealed FGF signaling as a consistently dysregulated pathway in both perichondrial and chondrocyte cells. The importance of FGF signaling in endochondral ossification is well supported, and mutations in FGFs and FGFRs result in various chondrodysplasias in humans (34). Increased FGFR2 activation and FGF9 and -18 stimulations in mouse models result in decreased bone growth characterized by decreased proliferative zones and no change in hypertrophic zone length, similar to our findings in the *Aga2^+/–^* mouse (46, 117, 118). Additionally, FGF9 and -18 are known to activate isoforms of FGFR2 (119). Increased FGF21 expression, which we observed in several *Aga2^+/–^* perichondrial and prehypertrophic chondrocyte clusters, has also been associated with the inhibition of chondrocyte proliferation due to reduced growth hormone binding (120). There is no previous work connecting FGF signaling to the OI phenotype; therefore, our single-cell analysis reveals FGF signaling as a possible treatment target that affects not only chondrocyte maturation but also progenitor cell function and communication.

There are limitations to our study. First, we only analyzed a single time point within OI growth plate development at P5 before formation of the secondary ossification center. Growth plate development is a highly dynamic process that changes as an organism matures. Therefore, additional time points may reveal further alterations in endochondral development. Second, upregulation of RNA expression does not necessarily translate to upregulation of protein, and we were cautious with our interpretation of pathway upregulation. Ideally, pathway activation must be confirmed via protein analysis. However, with the heterogeneity of expression demonstrated even within resting and differentiating chondrocyte zones, it would be a challenge to separate activation in differing cell types. Spatial proteomics technology is progressing at a fast pace and could be used in future studies to inform of changes at the protein level.

Despite limitations, our work has revealed dysregulation of chondrocyte differentiation throughout the *Aga2^+/–^* growth plate, and not only in cells expressing high levels of type I collagen, such as the perichondrium. Our data also indicate that *Sox9* and FGF signaling activation are drivers behind OI growth plate abnormalities in addition to or as a consequence of ER stress. Finally, this study provides insights into the gene expression heterogeneity of P5 mouse cartilage growth plate, as well as multiple cellular abnormalities in the *Aga2^+/–^* growth plate that contribute to disrupted endochondral ossification with subsequent effects on growth and bone (18).

## Methods

### Histological analysis.

Paraffin-embedded distal femoral growth plates from P8 mice were sectioned at 5 μm and then stained for collagen via Picrosirius red staining. Briefly, following deparaffinization, slides were stained for 30 minutes using Picrosirius red solution: 0.1% Direct Red 80 (MilliporeSigma, 43665)/Saturated Picric Acid (MilliporeSigma, P6744). Slides were washed in running tap water for 5 minutes and then counterstained with Mayer’s Hematoxylin for 20 seconds. Slides were again washed in tap water and then dehydrated and mounted.

For immunohistochemistry, paraffin sections were boiled for 20 minutes in Antigen Unmasking Solution (Vector) and stained for collagen 1 (Kerafast catalog ENH018-FP) using Goat Anti-Rabbit IgG Antibody (H+L), Biotinylated, R.T.U (Vector catalog BP-9100-50); Streptavidin, Peroxidase, R.T.U. (Vector catalog SA-5704-100); and ImmPACT DAB HRP Substrate (Vector catalog SK-4105). IHC experiments were performed with at least 3 biological replicates and 4 sections per replicate. The beginning of the proliferative zone was designated as when cells began forming columns of at least 3 cells and ending when cells began to enlarge where the hypertrophy began. The end of the hypertrophic zone was designated as when bone marrow and trabecular bone were visible. Measurements were taken from the middle of the growth plate.

### ScRNA-Seq analysis.

Initial data processing was completed using the Cell Ranger pipeline (10x Genomics) for demultiplexing, barcode assignment, and unique molecular identifier quantification. Downstream analyses were performed using Seurat V4. Cells with >6,000 and <200 expressed genes as well as >10% mitochondrial transcripts were excluded. Data normalization was performed using the SCTransform normalization method and integrated. Differential expression profiles derived from normalized RNA counts determined to be specific to the perichondrium or growth plate chondrocytes were analyzed using EnrichR and Database for Annotation, Visualization and Integrated Discovery for Gene Ontology and KEGG pathway analyses (121). Cell communication analysis was performed using the Nichenetr package, specifically the Differential NicheNet extension, which uses the differential expression between conditions and ligand-receptor pairs for prioritization (122).

### RNAscope.

We performed RNAscope analysis on cartilage samples fixed in 4% paraformaldehyde overnight followed by paraffin embedding. Cartilage sections were probed using the RNAscope Multiplex Fluorescent V2 assay from ACDBio. The standard protocol was followed with some modifications. Prior to deparaffinization, slides were baked at 60°C for 2 hours to encourage tissue adherence to the slides. Following deparaffinization, slides were again baked at 60°C for 30 minutes. Additionally, antigen retrieval and protease treatment steps were replaced with incubation using a custom reagent provided by ACDBio for 15 minutes at room temperature. RNAscope-processed slides were imaged at 20× original magnification using the Echo Revolution microscope, and images were processed using Adobe Photoshop. Probes used were Col1a1 (catalog 319371), Fgf9 (catalog 499811), Fgf18 (catalog 495421), Fgf21 (catalog 460931).

### Statistics.

Growth plate data were acquired using ImageJ (NIH) and analyzed using GraphPad Prism using the Student’s *t* test (2 tailed) to determine significance. Data represent mean ± SEM. scRNA-Seq data were analyzed, visualized, and statistically compared using R and the Seurat V4 package. *P* < 0.05 was considered significant.

### Study approval.

All animal studies were performed under protocols reviewed and approved by IACUC at the University of California, Los Angeles, and the Animal Research Committee, which is an independent research review committee mandated by the Animal Welfare Act and the PHS Policy on Humane Care and Use of Laboratory Animals.

### Data availability.

scRNA-Seq data sets have been deposited in the National Center for Biotechnology Information’s Gene Expression Omnibus (accession number GSE231795). Supporting analytic code can be accessed on GitHub (https://github.com/jenzieba/Cartilage_ScRNAseq; commit ID c79e0b9).

All data associated with this study are present in the paper, Supporting Data Values file, Supplemental Data 1–5, or public repositories. Please contact the corresponding author for reagents and resources generated in this study.

Further methods details may be found in Supplemental Methods.

## Author contributions

DK, JZ, DHC, and LN conceived and designed the experiments. JZ, LN, JHM, and SW performed the experiments. JZ and DW analyzed the data. DK and AK contributed reagents/materials/analysis tools. DK and JZ wrote the paper.

## Supplementary Material

Supplemental data

Supplemental data set 1

Supplemental data set 2

Supplemental data set 3

Supplemental data set 4

Supplemental data set 5

Supporting data values

## Version 1 10/05/2023

In-Press Preview

## Version 2 11/08/2023

Electronic publication
