# Supplementary material for: Altered Sox9 and FGF signaling gene expression in Aga2 OI mice negatively affects linear growth
Source: JCI Insight. 2023 Nov 8;8(21):e171984. doi: 10.1172/jci.insight.171984 (PMC10721276; doi:10.1172/jci.insight.171984)
Supplement: Supplemental data [file jciinsight-8-171984-s143.pdf]

Supplemental methods:

Generation of Mice

Aga2$^{+/-}$ animals were received as a gift from the Jacobsen laboratory at Harvard Medical School in Boston, Massachusetts and maintained on a C57BL/6J background (18). The *Aga2*$^{+/-}$ colony was maintained through mutant alleles only passed through males. UCLA follows the PHS Policy that requires that all institutions base their animal care and use programs on the Guide for the Care and Use of Laboratory Animals, and that euthanasia be consistent with the American Veterinary Medical Association (AVMA) Guidelines on Euthanasia. The animals were monitored at least once daily and once during the weekend by personnel associated with this proposal, and by the veterinary and technical staff of the UCLA Department of Laboratory Animal Medicine. Fresh water and food were supplied for consumption as needed, and visibly unhealthy animals were removed immediately and euthanized by inhalation. Sentinel animals are used for detailed serological testing to ensure that the colony remains pathogen free.

Tissue collection, dissociation for single cell RNAseq

Proximal tibia and distal femur epiphyses from postnatal day 5 mouse pups were collected and digested with Collagenase I tissue removal solution (1% Collagenase I, 2.5% trypsin w/o EDTA, 30mM $Cacl_{2,}$ in HBSS) at 37ºC for 45 minutes to remove muscle tissue that remained following dissection. Cartilage samples were then incubated at 37ºC in Collagenase II single cell isolation solution (1% Collagenase II, 2.5% trypsin w/o EDTA, 30mM $Cacl_{2,}$ in HBSS) for 2-3 hours with brief vortexing every 10-15 minutes. Single cells were passed through a 40um cell strainer in PBS containing 0.04% BSA. Cell numbers and viability were quantified and only samples with great than 80% cell viability were used for subsequent cell encapsulation and library construction. Cells were encapsulated into emulsion droplets by Chromium Controller (10x Genomics) and libraries were constructed using the 10X Genomics Chromium Single Cell 3′ v3 reagent kit. cDNA libraries were sequenced in a Novaseq S2 sequencer using using 100-cycle paired-end reads, generating ~45,000 reads per cell with a total of ~10,000 cells per sample.

Cell type	Cell sub-type	Markers
Articular Cartilage	Art. Cart. #1	Prg4
	Art. Cart. #2	Prg4
	Art. Cart. #3	Prg4
Articular Chondrocytes	Art. Ch. #1	S100a6, s100a10
	Art. Ch. #2	Ucma, Sox9, Acan, Matn3, col6, col3, col12, Arf4
	Art. Ch. #3	Ucma, Sox9, Acan, Matn3, Rhob, Txnip, Tsc22d1
	Art. Ch. #4	Ucma, Sox9, Acan, Matn3, Bmp3, Nfat5, Rbp4
Resting Chondrocytes	Cfos+ RCh	Ucma, Sox9, Acan, Matn3, Bmp3, Meg3, Epyc
	Mia+ RCh	Ucma, Sox9, Acan, Matn3
	MT1+ RCh	Ucma, Sox9, Acan, Matn3
	MT2+ RCh	Ucma, Sox9, Acan, Matn3, Bmp3
	nonDiff RCh	Ucma, Sox9, Acan, Matn3, Grem1
	Sox8 RCh	Acan, Matn3, Sox8
	Barx2 RCh	Barx2, Neat1, Tgfbi
	Early Prolif. RCh	Ucma, Sox9, Acan, Matn3, Bmp3, Hspa1a, Hspa1b, MMP14
	Tgfbi+ Prehyp. Ch. #1	Mgp, Sox4, Nt5e, Tgfbi, Spp1
	Tgfbi+ Prehyp. Ch. #2	Mgp, Sox4, Nt5e, Tgfbi, Spp1
Proliferative Chondrocytes	Proliferative GPC #1	Sox9, Acan, Matn3, Prelp, Igf1r, Cdk1, Hmmr
	Proliferative GPC #2	Ucma, Sox9, Acan, Matn3, Hspa5, Herpud1, Cthrc
	Proliferative GPC #3	Gdf10, Sox9, Acan, Matn3, Prelp, Igf1r, Runx2
	Proliferative GPC #4	Ucma, Sox9, Acan, Matn3, Prdx2, Rpsa, Pclaf, Ppib
Pre/hypertrophic Chondrocytes	Pre/hypertrophic GPC #1	Atf3, Sox9, Gadd45g
	Pre/hypertrophic GPC #2	Ihh, Frzb1
	Pre/hypertrophic GPC #3	Ihh, Fgf21, histone genes
	Pre/hypertrophic GPC #4	Ihh, Cxcl10
	Pre/hypertrophic GPC #5	Ucma, Sox9, Acan, Matn3, Fdps
	Hypertrophic/terminal GPC	col10a1, Fgfr1, Gcnt1, Krt16, Plaur
Perichondrial	Prolif. Chondrogenic Precursor	Postn, Col3a1, Vcan, Col1a1, CDK1
	OB. Progenitors	Ctsk, Mmp2, Mmp13, Postn, Col3a1, Vcan, Col1a1
	Perichondrial #1	Postn, Col3a1, Vcan, Col1a1, Filip1l, Clec3b
	Perichondrial #2	Postn, Col3a1, Vcan, Col1a1, Tnn, Mthfd2, Eif4ebp1
	Perichondrial Diff.	Pthlh, Prrx1, Col1a1, Sox4
Others	Pericytes/endothelial	Notch3, Hes1, Sox4, Pecam1, Cdh5
	Platelets	Pf4

Supplementary table 1. Markers used to delineate between chondrocyte and perichondrial cluster subtypes.

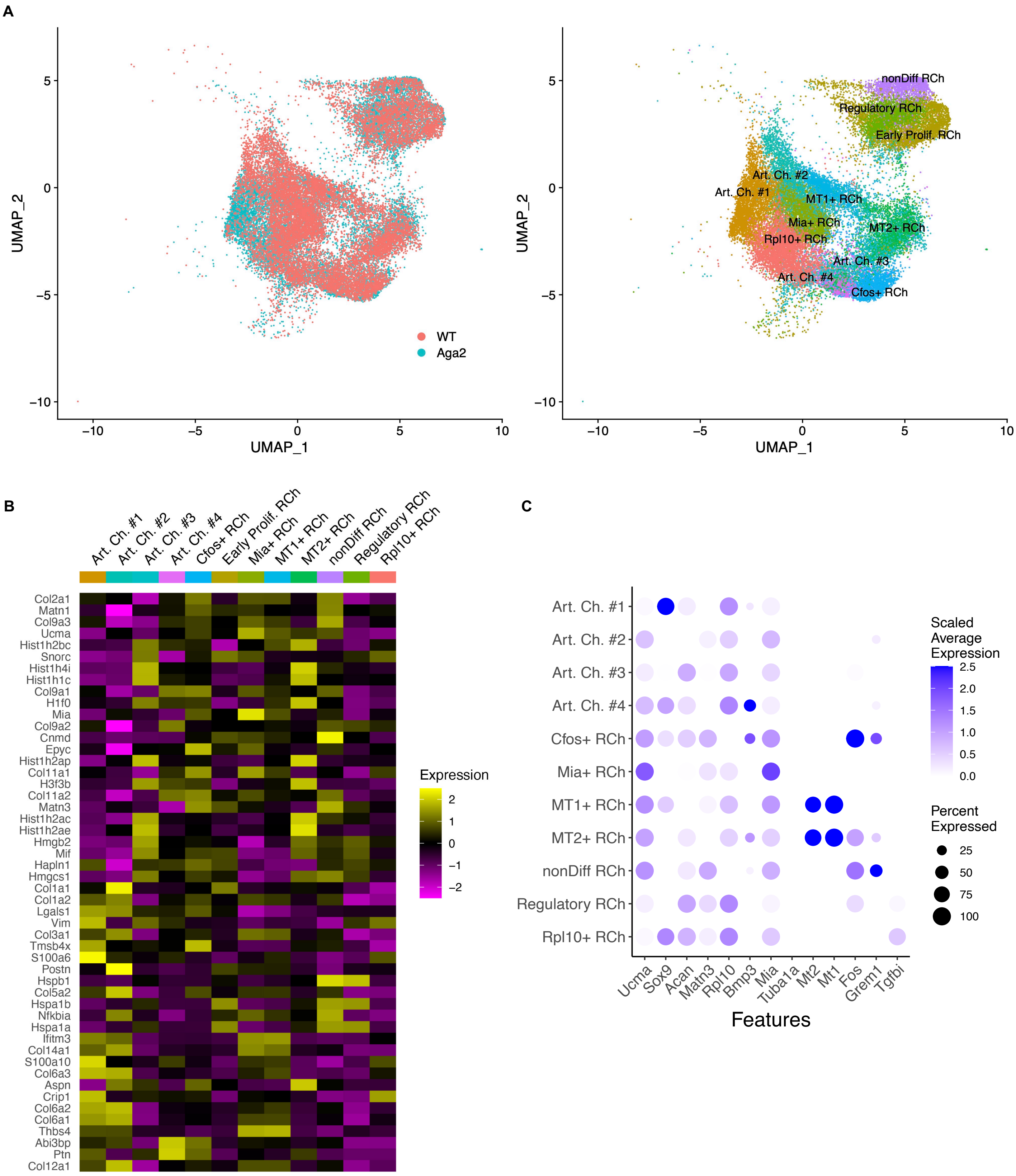

Supplementary figure S1. Single cell RNAseq analysis of resting chondrocyte cluster subset. (A) Cell clusters from scRNA-seq analysis visualized by Uniform Manifold Approximation and Projection (UMAP). Left: Cells that correspond to WT (pink) and *Aga2*$^{+/-}$ (blue) resting chondrocytes. Right: Colors indicate clusters of various cell types. N = 5 for each genotype. (B) Heatmap showing top differentially expressed genes in each cluster within the subset. (C) Dot plot showing the expression of selected markers of various cell types. Dot size represents the % of cells expressing a specific marker, while the intensity of color indicates the average expression level for that gene, in that cluster.

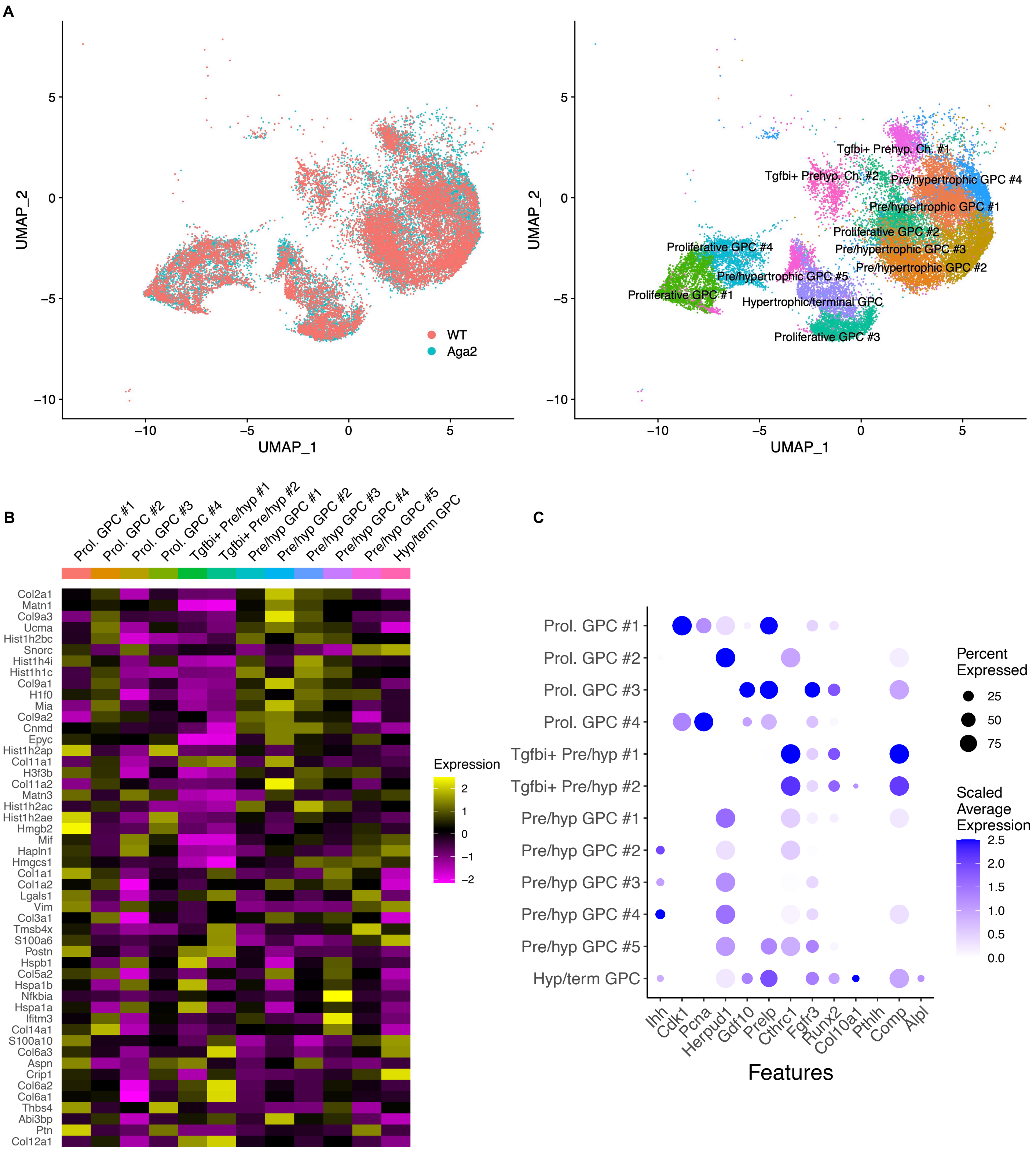

Supplementary figure S2. Single cell RNAseq analysis of differentiating chondrocyte cluster subset. (A) Cell clusters from scRNA-seq analysis visualized by Uniform Manifold Approximation and Projection (UMAP). Left: Cells that correspond to WT (pink) and *Aga2*$^{+/-}$ (blue) differentiating chondrocytes. Right: Colors indicate clusters of various cell types. N = 5 for each genotype. (B) Heatmap showing top differentially expressed genes in each cluster within the subset. (C) Dot plot showing the expression of selected markers of various cell types. Dot size represents the % of cells expressing a specific marker, while the intensity of color indicates the average expression level for that gene, in that cluster.

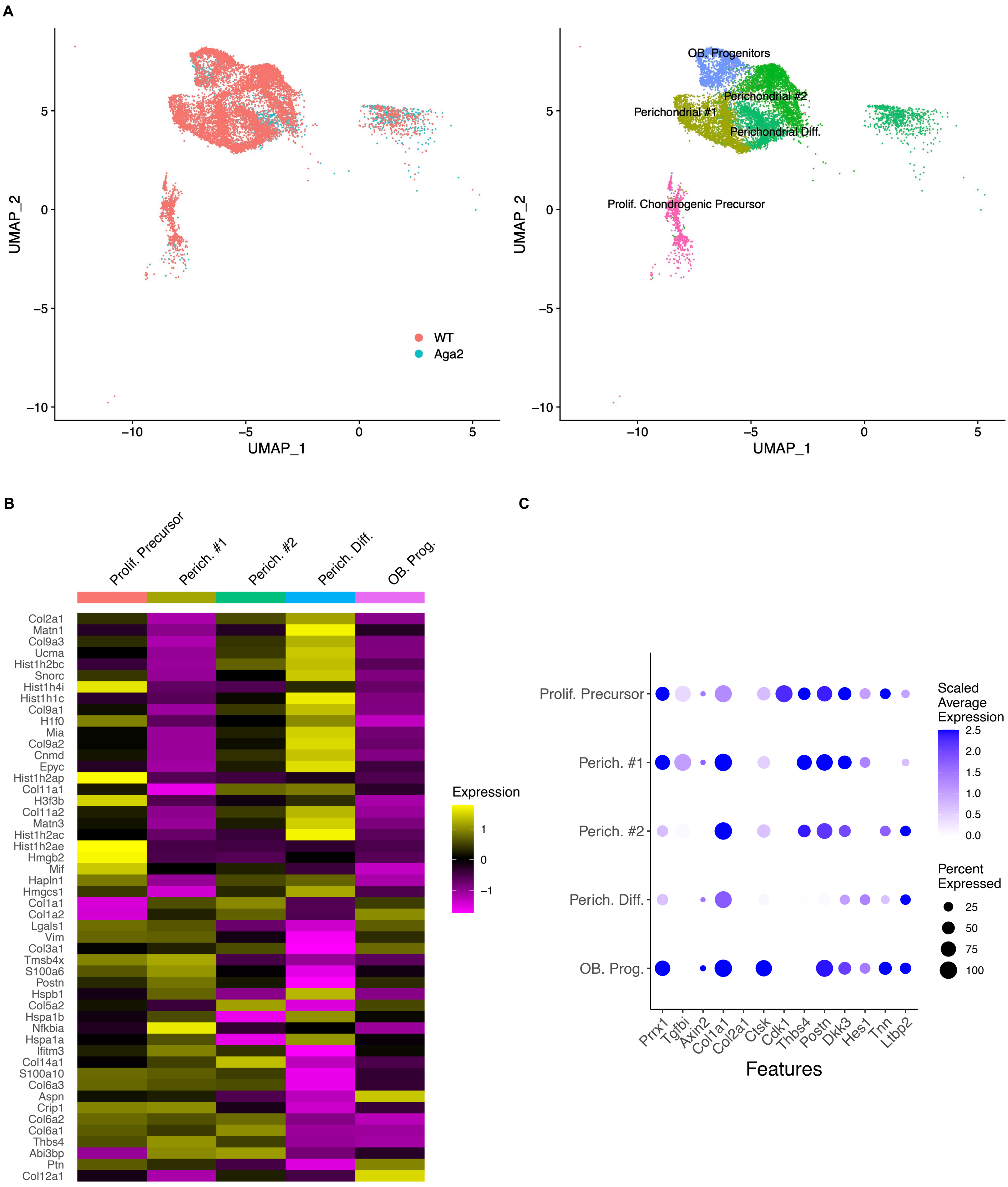

Supplementary figure S3. Single cell RNAseq analysis of perichondrial cell cluster subset. (A) Cell clusters from scRNA-seq analysis visualized by Uniform Manifold Approximation and Projection (UMAP). Left: Cells that correspond to WT (pink) and *Aga2*$^{+/-}$ (blue) perichondrial cells. Right: Colors indicate clusters of various cell types. N = 5 for each genotype. (B) Heatmap showing top differentially expressed genes in each cluster within the subset. (C) Dot plot showing the expression of selected markers of various cell types. Dot size represents the % of cells expressing a specific marker, while the intensity of color indicates the average expression level for that gene, in that cluster.

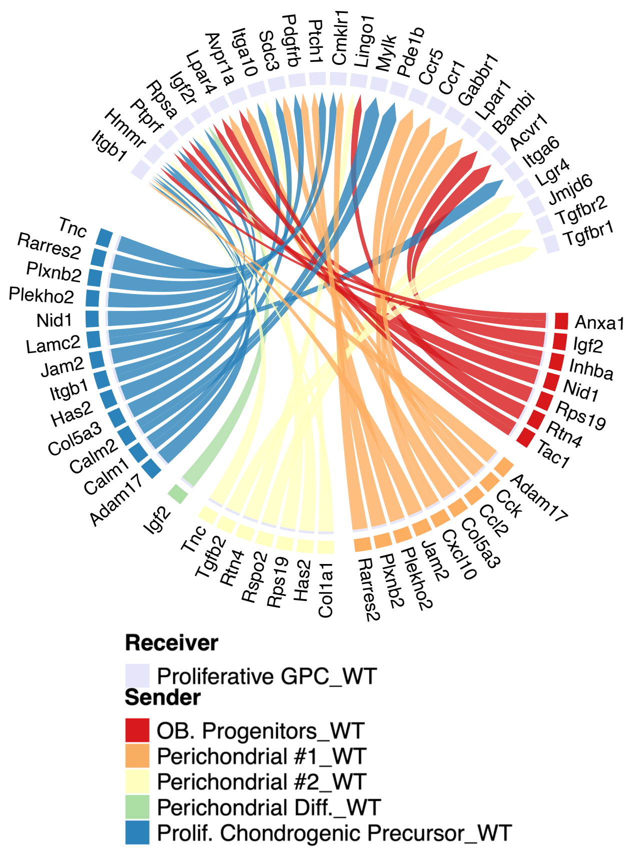

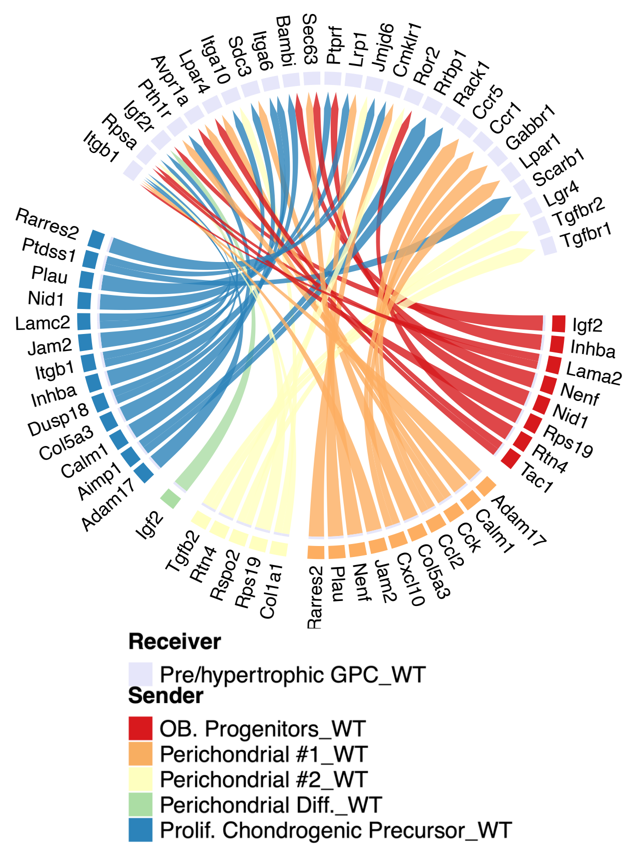

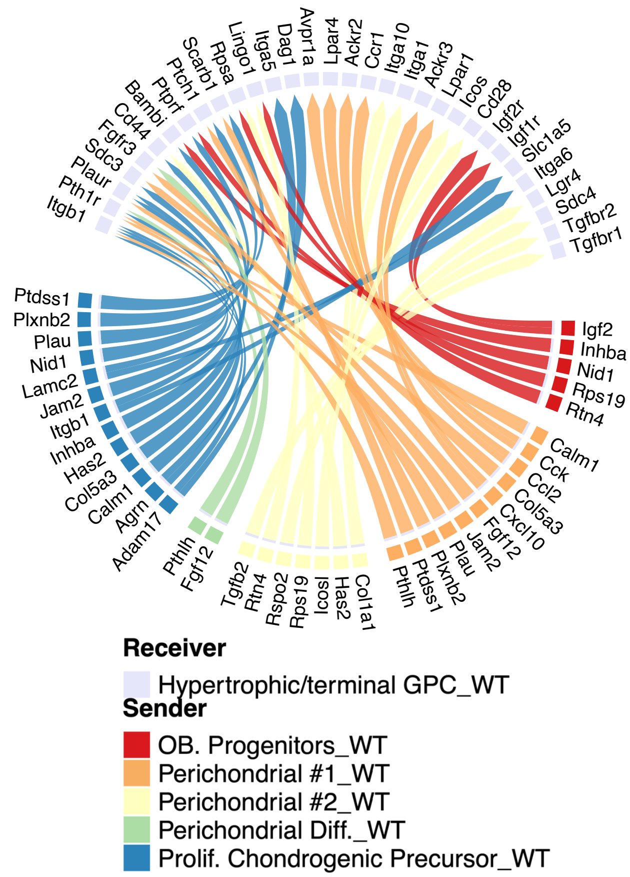

Supplementary figure S4. Downregulated cell communication between progenitors and growth plate cells in *Aga2*$^{+/-}$ cartilage. Circos plots showing inferred upregulated cell communication via Nichenet analysis. Top left: proliferative chondrocytes, middleright: pre/hypertrophic chondrocytes, bottom left: hypertophic/terminal chondrocytes. Differentiating 'receiver' chondrocytes with increased expression of signaling receptors and downstream targets are connected to the perichondrial 'sender' cell types expressing ligands predicted to promote this response. Ligands expressed by the same cell population are colored the same.

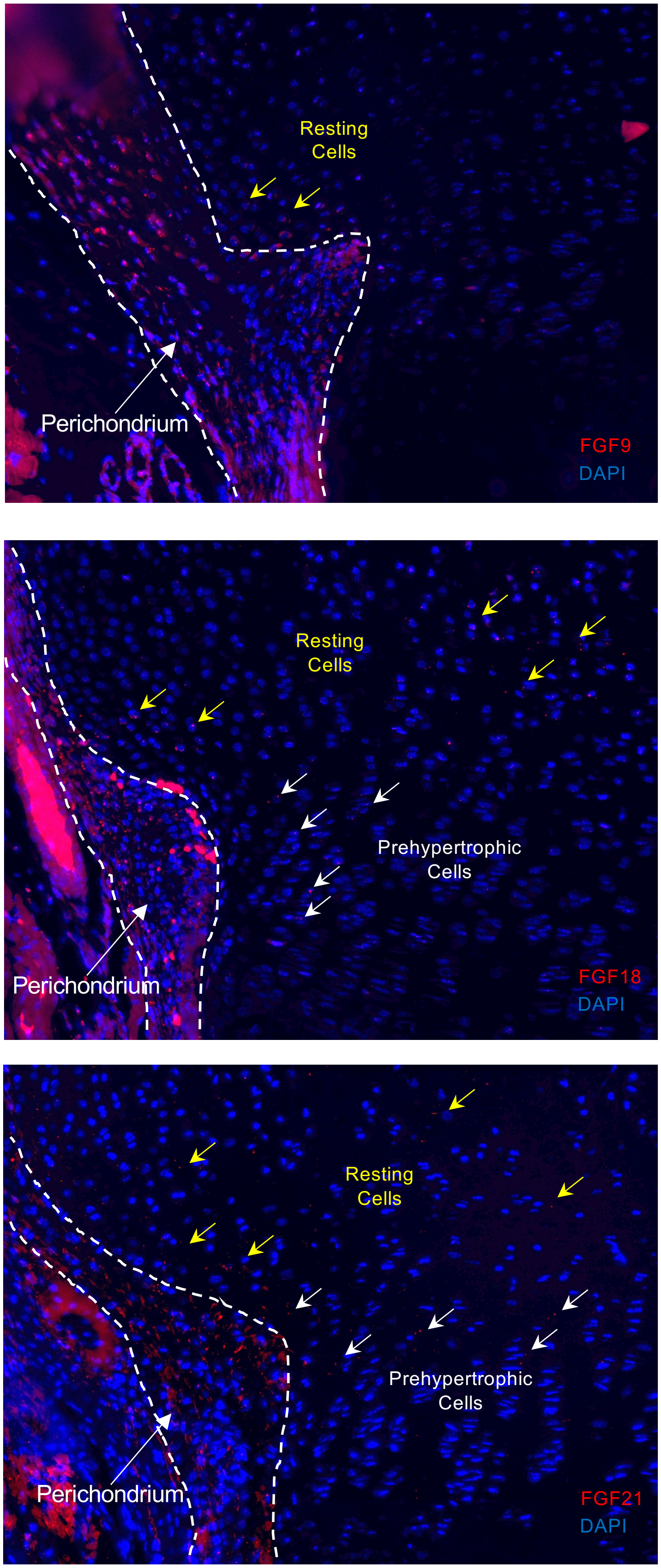

Supplementary figure S5. FGF ligands are expressed in perichondrium and nearby chondrocytes. RNAscope for FGF9, FGF18, and FGF21 (red) co-stained with Dapi (blue.) Yellow arrows point to RNAscope signal in resting zone chondrocytes, white arrows point to RNAscope signal in prehypertrophic zone chondrocytes. Dashed line delineates perichondrium. N = 3.
